# Ascitic fluid shear stress in concert with hepatocyte growth factor drive stemness and chemoresistance of ovarian cancer cells via the c-Met-PI3K/Akt-miR-199a-3p signaling pathway

**DOI:** 10.1038/s41419-022-04976-6

**Published:** 2022-06-08

**Authors:** Ayon A. Hassan, Margarita Artemenko, Maggie K. S. Tang, Zeyu Shi, Lin-Yu Chen, Hung-Cheng Lai, Zhenyu Yang, Ho-Cheung Shum, Alice S. T. Wong

**Affiliations:** 1 School of Biological Sciences, University of Hong Kong, Pok Fu Lam, Pokfulam Road, Hong Kong; 2 Laboratory for Synthetic Chemistry and Chemical Biology Limited, 17 W, Hong Kong Science and Technology Parks, Shatin, New Territories Hong Kong; 3 Department of Obstetrics and Gynecology, Shuang Ho Hospital, Taipei Medical University, Taipei, 23561 Taiwan; 4 Department of Obstetrics and Gynecology, School of Medicine, College of Medicine, Taipei Medical University, Taipei, Taiwan; 5 Department of Mechanical Engineering, University of Hong Kong, Pok Fu Lam, Pokfulam Road, Hong Kong; 6 Advanced Biomedical Instrumentation Centre, Hong Kong Science Park, Shatin, New Territories Hong Kong

**Keywords:** Ovarian cancer, Cell signalling

## Abstract

Overcoming drug resistance is an inevitable challenge to the success of cancer treatment. Recently, in ovarian cancer, a highly chemoresistant tumor, we demonstrated an important role of shear stress in stem-like phenotype and chemoresistance using a three-dimensional microfluidic device, which most closely mimics tumor behavior. Here, we examined a new mechanosensitive microRNA—miR-199a-3p. Unlike most key microRNA biogenesis in static conditions, we found that Dicer, Drosha, and Exportin 5 were not involved in regulating miR-199a-3p under ascitic fluid shear stress (0.02 dynes/cm^2^). We further showed that hepatocyte growth factor (HGF), but not other ascitic cytokines/growth factors such as epidermal growth factor and tumor necrosis factor α or hypoxia, could transcriptionally downregulate miR-199a-3p through its primary transcript miR-199a-1 and not miR-199a-2. Shear stress in the presence of HGF resulted in a concerted effect via a specific c-Met/PI3K/Akt signaling axis through a positive feedback loop, thereby driving cancer stemness and drug resistance. We also showed that miR-199a-3p expression was inversely correlated with enhanced drug resistance properties in chemoresistant ovarian cancer lines. Patients with low miR-199a-3p expression were more resistant to platinum with a significantly poor prognosis. miR-199a-3p mimic significantly suppressed ovarian tumor metastasis and its co-targeting in combination with cisplatin or paclitaxel further decreased the peritoneal dissemination of ovarian cancer in mice. These findings unravel how biophysical and biochemical cues regulate miR-199a-3p and is important in chemoresistance. miR-199a-3p mimics may serve as a novel targeted therapy for effective chemosensitization.

## Introduction

The tumor microenvironment plays a critical role in tumor progression and therapeutic response [1]. Exciting new studies have begun to unravel that, besides cellular components, acellular factors especially mechanical stimuli are involved in modulating malignant gene expression and/or cellular function of tumor cells [2]. However, because a dynamic flow system is more difficult to manipulate and study as compared to a static system, the tumor/stem-like phenotype under fluid flow has not been thoroughly investigated and remains poorly understood. This also highlights some limitations in the current in vitro platforms for drug screening. Several systems have been developed to study the effect of fluid flow on cultured cells, ranging from large bioreactors to compact parallel plate devices [3]. However, they suffer from inability to manipulate fluid flow and generate low shear stress comparable to that in the peritoneal cavity [4].

Ovarian cancer is the most lethal gynecological malignancy with around two-thirds of the patients diagnosed at an advanced stage (stage III or IV) [5]. Peritoneal ascites is a common complication of advanced/metastatic ovarian cancer, which constitutes a unique mechanical microenvironment where tumor cells experience at least 10-fold lower shear stress level than in the blood [6], suggesting that peritoneal dissemination may involve significantly different molecular mechanism from that of blood-borne metastasis. Therapeutic approaches for malignant ascites are primarily palliative in nature, resulting in a 5-year survival rate of around 1 in 4 patients [7, 8]. The role for the fluid shear stress in peritoneal metastasis however remains elusive.

The standard protocol of treatment of advanced-stage ovarian cancer is maximal cytoreductive surgery followed by taxane/platinum chemotherapy [9]. While most patients exhibit a good initial response, majority (over 75%) of the patients eventually experience relapse with resistance to additional treatments and succumb to the disease [10]. This makes ovarian cancer a very difficult to treat disease, with little to no improvement in patient survival over the last few decades [11, 12]. It is therefore of utmost importance to delineate the molecular mechanism of chemoresistance.

MicroRNAs (miRNAs) are small non-coding RNAs that are known to regulate gene expression by translational repression or degradation of messenger RNAs (mRNAs) [13]. The primary miRNA (pri-miRNA) is cleaved by RNase III Drosha into precursor miRNA (pre-miRNA) [14, 15]. Exportin-5 transports the pre-miR from the nucleus to the cytoplasm for Dicer-mediated processing into functionally mature miRNA [16, 17]. Dysregulated miRNAs are known to act as tumor suppressors and oncogenes in ovarian cancer [18–22]. Using a three-dimensional microfluidic platform, we previously identified that shear stress from ascitic current induces chemoresistance and stemness in ovarian cancer cells [23]. Importantly, this was associated with downregulation of miR-199a-3p, suggesting a previously unknown mechanosensitive property of this miRNA. Yet, the mechanism by which ascitic shear stress regulates miR-199a-3p remains unknown.

In this study, we demonstrate a concerted action of biophysical and biochemical cues in regulating miR-199a-3p through a c-Met/PI3K/Akt signaling axis in a positive loop. We further provide evidence for its importance in chemoresistance and that targeting miR-199a-3p could represent an effective strategy to resensitize ovarian tumor cells to chemotherapy.

## Results

### Shear stress transcriptionally downregulates miR-199a-3p through miR-199a-1

**Fig. 1:**
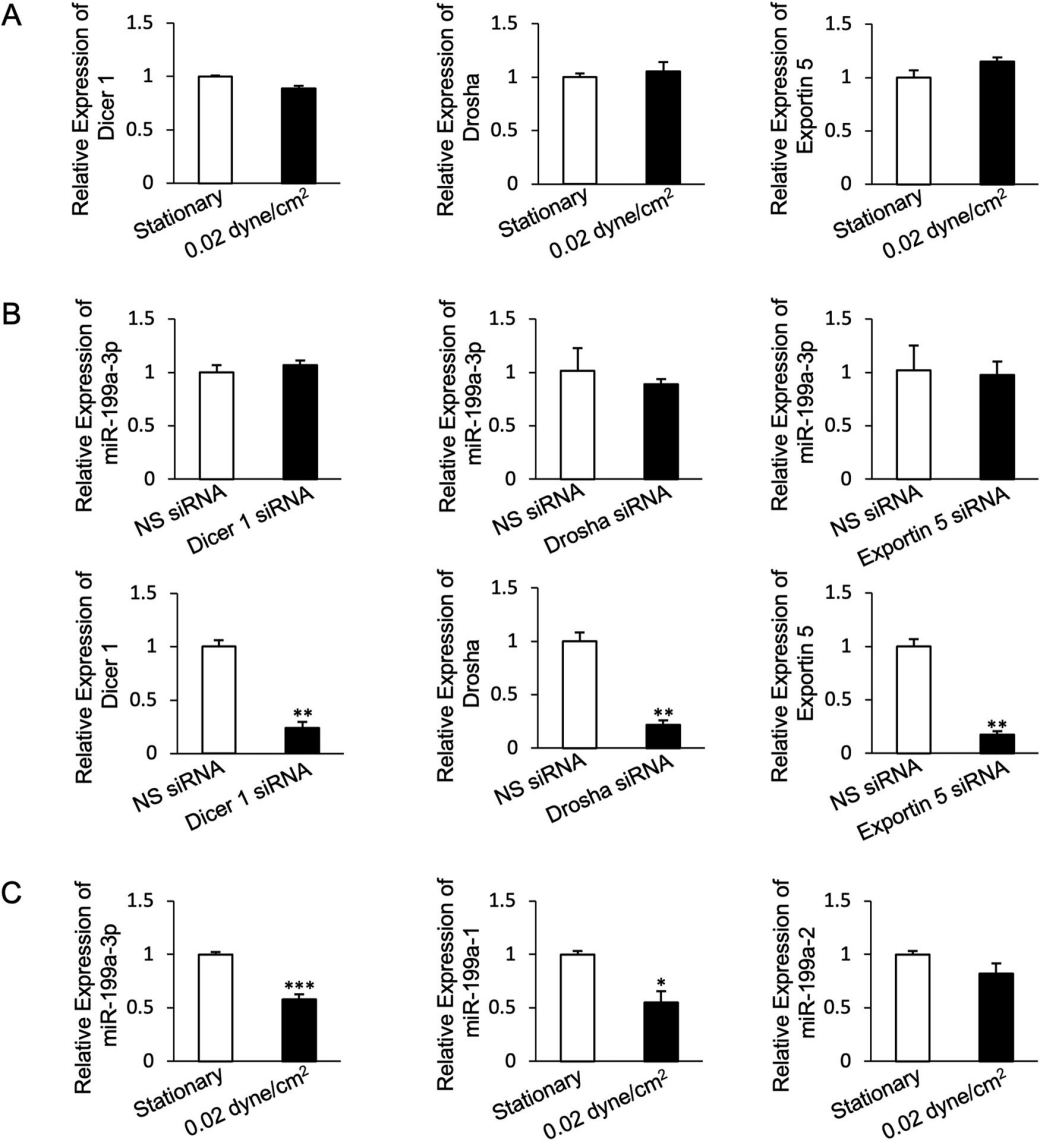
miR-199a-3p is transcriptionally regulated by shear stress. **A** Expression of miRNA biogenesis regulators Dicer 1, Drosha, and Exportin 5 under shear stress. **B** Expression of miR-199a-3p upon knockdown of Dicer 1, Drosha, and Exportin 5. **C** Expression of miR-199a-3p and its primary transcripts miR-199a-1 and miR-199a-2 under shear stress. Bar graph values are mean ± SD, *n* = 3. *T*-test. *P < 0.05, **P < 0.005, ***P < 0.0005.

To determine how shear stress may regulate miR-199a-3p expression, we first assessed its effect on Dicer, Drosha, and Exportin 5, the major miRNA biogenesis regulators. However, we found little or no change in gene expression of Dicer, Drosha, and Exportin 5 under shear stress (Fig. 1A). Knocking down Dicer, Drosha, or Exportin 5 also did not affect miR-199a-3p expression (Fig. 1B). We then assessed other aspects of miR-199a-3p biogenesis as a result of shear stress. In the human genome, two genes encode mature miR-199a-3p, MIR199A1 on chromosome 19 and MIR199A2 on chromosome 1 [24]. While we found no effect of shear stress on miR-199a-2, the expression of miR-199a-1 was significantly downregulated by shear stress (Fig. 1C), suggesting a specific role for shear stress in downregulating miR-199a-3p via a transcriptional mechanism through miR-199a-1.

### HGF downregulates miR-199a-3p through miR-199a-1

**Fig. 2:**
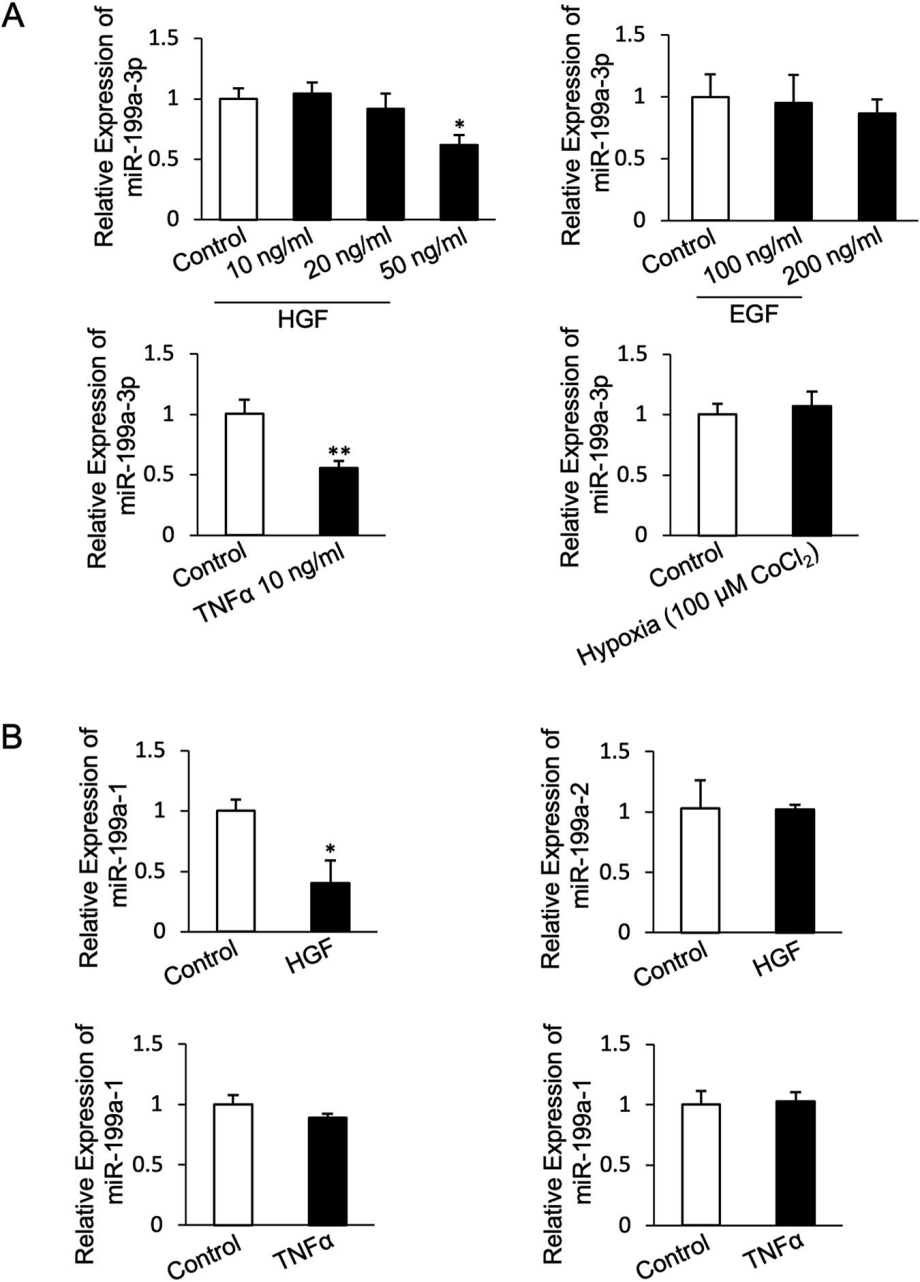
Hepatocyte growth factor transcriptionally regulates miR-199a-3p. **A** Expression of miR-199a-3p upon HGF treatment (10–50 ng/ml), EGF treatment (100–200 ng/ml), TNFα (10 ng/ml) treatment, and hypoxia (100 μM CoCl_2_) for 24 h. **B** Expression of primary transcripts miR-199a-1 and miR-199a-2 upon 50 ng/ml HGF and 10 ng/ml TNFα treatment for 24 h. Bar graph values are mean ± SD, *n* = 3. *T*-test. *P < 0.05, **P < 0.005.

**Fig. 3:**
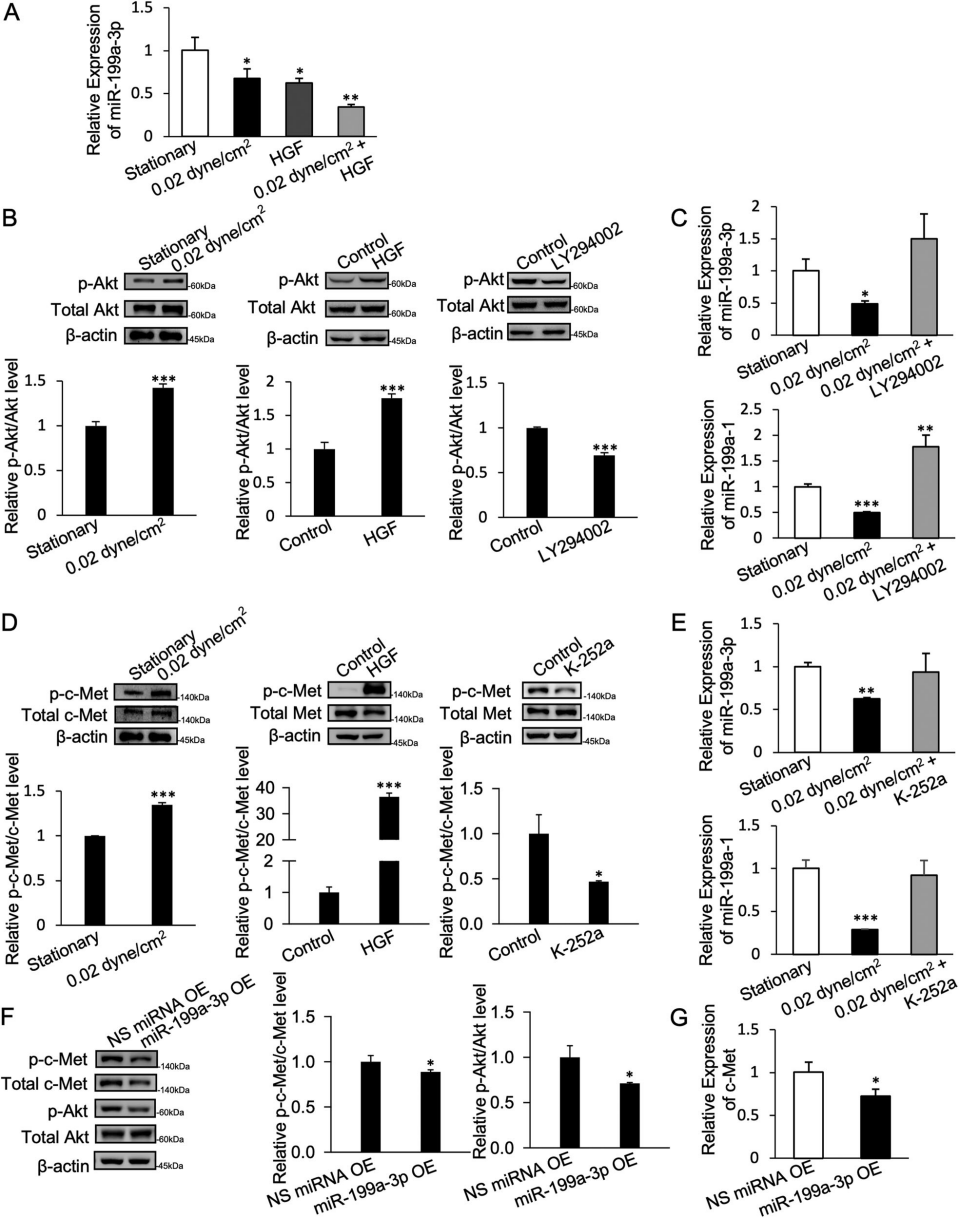
c-Met/PI3K/Akt pathway regulates miR-199a-3p through a positive feedback loop. **A** Expression of miR-199a-3p under shear stress, HGF treatment, and combination of shear stress and HGF. **B** Expression of p-Akt and total Akt under shear stress, HGF, and LY 294002 treatment. **C** Expression of miR-199a-3p and miR-199a-1 when treated with LY 294002 under shear stress. **D** Expression of p-c-Met and total c-Met under shear stress, HGF, and c-Met inhibitor K-252a. **E** Expression of miR-199a-3p and miR-199a-1 when treated with K-252a under shear stress. **F** Expression of p-c-Met, total c-Met, p-Akt, total-Akt, and **G**. c-Met mRNA in miR-199a-3p overexpressing cells. Bar graph values are mean ± SD, *n* = 3 for q-PCR and mean ± SD of three replicate measures of respective bands for Western Blot quantification. *T*-test. *P < 0.05, **P < 0.005, ***P < 0.0005.

Malignant ascites harbors significantly higher levels of certain cytokines and growth factors [25]. We examined the effects of hepatocyte growth factor (HGF), epidermal growth factor (EGF), and tumor necrosis factor alpha (TNFα), which are particularly relevant to the progression of ovarian cancer [26–28]. Interestingly, HGF and TNFα significantly reduced miR-199a-3p levels, whereas EGF had no effect (Fig. 2A). Furthermore, we found a decrease in the expression of primary miR-199a-1 upon HGF treatment, but not with that of TNFα treatment (Fig. 2B). None of the growth factors/cytokines affected miR-199a-2 expression. Using hypoxia mimetics (CoCl_2_), we also found no effect of hypoxia on miR-199a-3p (Fig. 2A). More importantly, cotreatment with shear stress and HGF resulted in significantly more reduction of miR-199a-3p expression than shear stress or HGF treatment alone (Fig. 3A). These data suggest that HGF and shear stress may regulate miR-199a-3p in an additive manner in malignant ascites.

### Physiologically representative shear stress and HGF downregulate miR-199a-3p via c-Met/PI3K/Akt through a positive feedback loop

To delineate the signaling pathway that mediates the miR-199a-3p downregulation, we tested a set of small molecule inhibitors that specifically target phosphoinositide 3-kinase (PI3K), IκB kinase (IKK), and mitogen-activated protein kinase (MEK), which are kinases commonly activated by HGF and TNFα. We found that only inhibitors of the PI3K (LY294002) and IKK (BAY11-7082) could upregulate miR-199a-3p. In contrast, the MEK inhibitor PD98059 did not affect miR-199a-3p expression (Supplementary Fig. 1). Moreover, Akt-specific siRNA could also upregulate miR-199a-3p. LY294002-mediated upregulation of miR-199a-3p was found to be through its primary transcript miR-199a-1, whereas Bay11-7082 did not affect primary miR-199a-1.

The HGF receptor c-Met is commonly overexpressed in ovarian cancer [29]. We further examined whether shear stress may mediate its mechanotransduction through c-Met. Our results showed that shear stress was able to activate c-Met and PI3K/Akt signaling pathway, whereas inhibitors against c-Met (K-252a), as well as PI3K/Akt (LY294002), could abrogate the downregulation of miR-199a-3p and miR-199a-1 under shear stress (Fig. 3B–E). Moreover, overexpression of miR-199a-3p could downregulate the expression of c-Met mRNA and reduce levels of phosphorylated Akt (Fig. 3F, G), indicating that the c-Met/PI3K/Akt/miR-199a-3p axis forms a positive feedback loop.

### miR-199a-3p regulates stemness and drug resistance in ovarian cancer cells

**Fig. 4:**
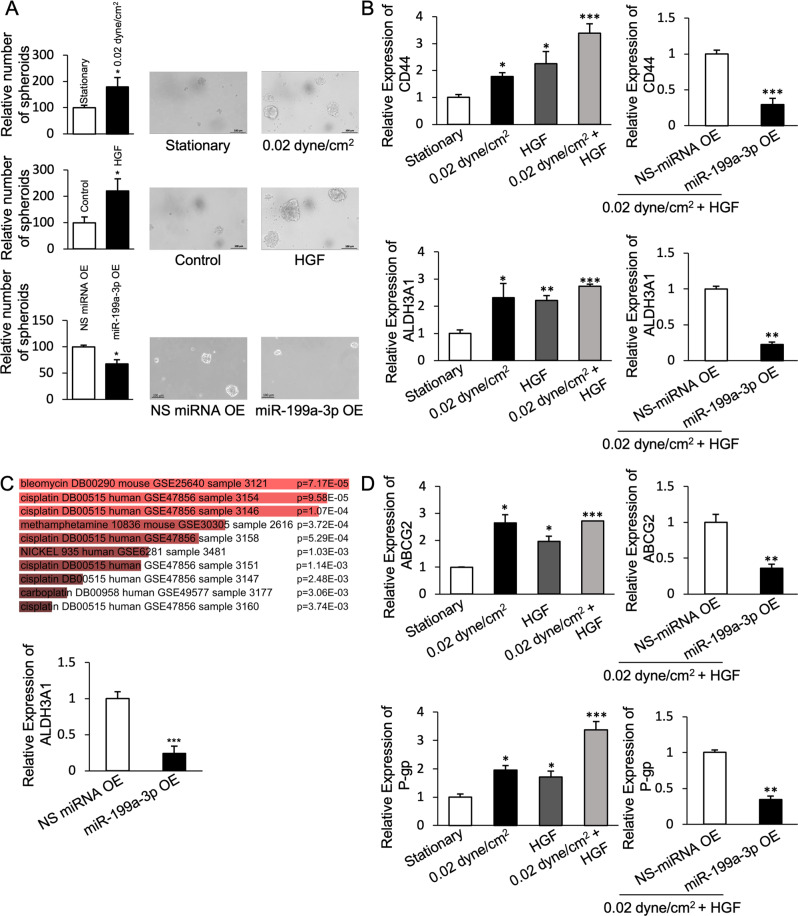
miR-199a-3p enhances stemness in ovarian cancer cells. **A** Relative number of spheroids formed under shear stress and HGF treatment, and in miR-199a-3p overexpressing cells. Scale bar, 100 μm. **B** Expression of CD44, and ALDH3A1 under shear stress an HGF combined treatment, and their expression in miR-199a-3p overexpressing cells. **C** List of drug metabolism pathways in EnrichR database correlated with shear stress upregulated genes sets from whole cell RNA sequencing and expression of ALDH3A1 in miR-199a-3p overexpressing cells. **D** Expression of ABCG2 and P-gp under shear stress and HGF combined treatment, and their expression in miR-199a-3p overexpressing cells. Bar graph values are mean ± SD, *n* = 3. *T*-test. *P < 0.05, **P < 0.005.

Cancer stem cells (CSCs) are a subpopulation of tumor cells postulated to be responsible for tumor initiation and ultimately cause disease relapse. We observed that under conditions of shear stress or HGF treatment, where miR-199a-3p is downregulated, ovarian cancer cells were able to form more spheroids under stem cell-selective conditions (Fig. 4A), indicating an association between miR-199a-3p and stemness. Overexpression of miR-199a-3p diminished the ability of ovarian cancer cells to form spheroids (Fig. 4A). Further, transcriptomic analysis of shear stress-treated cells revealed that genes over-represented under shear stress are involved in the metabolism of cytotoxic agents that are used to treat ovarian cancer patients in the clinic, including cisplatin, carboplatin, and bleomycin (Fig. 4C). Among the overexpressed genes, we found ALDH3, which is a marker of stemness (and chemoresistance) in ovarian cancer (Supplementary Table 1). Co-treatment of shear stress and HGF resulted in upregulation of ALDH3 and ovarian cancer stemness marker CD44, which was abrogated in the presence of miR-199a-3p overexpression (Fig. 4B).

**Fig. 5:**
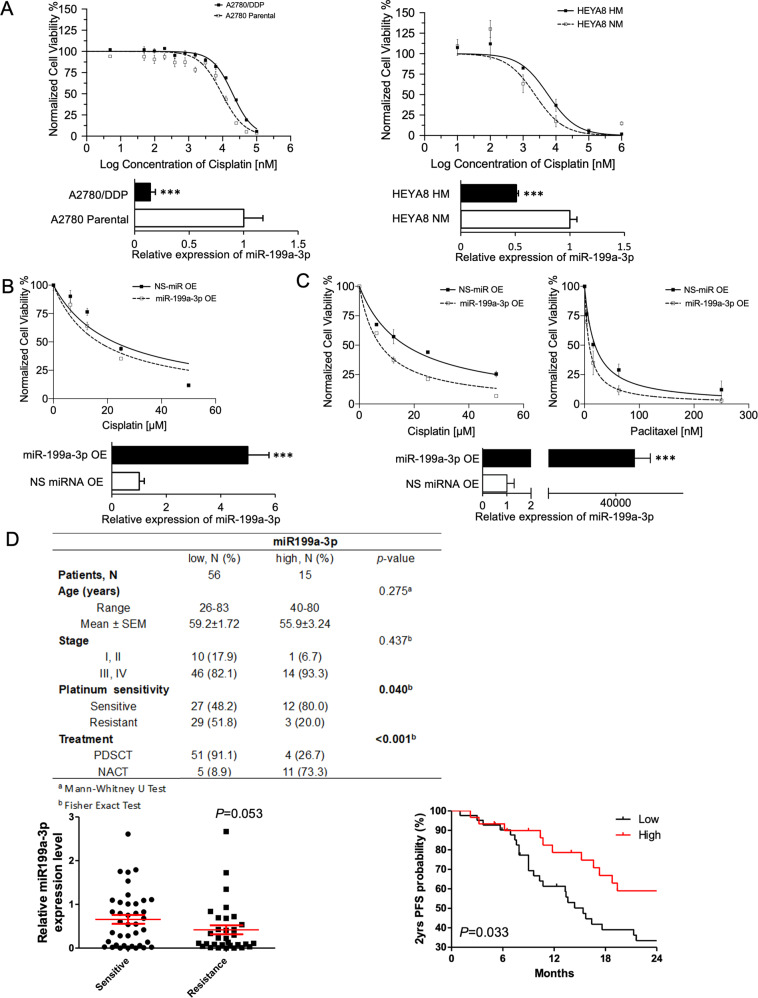
miR-199a-3p enhances chemosensitivity and stemness in ovarian cancer cells. **A** Dose–response curve of A2780 Parental vs. A2780/DDP cells (left), and HEYA8 HM vs. HEYA8 NM cells (right) against cisplatin. **B** Dose–response curve of miR-199a-3p-overexpressing A2780/DDP cells (left) against cisplatin and **C** against cisplatin/paclitaxel for SKOV-3 cells (right). The miR-199a-3p-overexpressing SKOV-3 cells were divided into two groups: cisplatin and paclitaxel treatment. **D** Expression of miR-199a-3p in chemosensitive vs. chemoresistant patient tumors and Kaplan–Meier Survival analysis for patient stratified by miR-199a-3p expression, and clinicopathological table of patients. Bar graph values are mean ± SD, *n* = 3. *T*-test. *P < 0.05, **P < 0.005. Statistical analysis for patient samples is mentioned in clinicopathological table.

CSCs are responsible for the tumor initiation and recurrence after chemotherapy. Treatment with shear stress in the presence of HGF resulted in downregulation of drug efflux protein-coding genes ABCG2 and P-gp, but not in miR-199a-3p overexpressing cells (Fig. 4D). In order to assess the potential role of miR-199a-3p in chemoresistance, we evaluated the expression of miR-199a-3p in isogenic ovarian cancer cell lines that differ in chemosensitivity. Cisplatin-resistant A2780 (A2780/DDP) cells exhibited significantly lower expression of miR-199a-3p compared to cisplatin-sensitive A2780 (Fig. 5A). Similarly, highly metastatic HEYA8 (HEYA8 HM) cells had lower expression of miR-199a-3p and were more resistant to cisplatin compared to non-metastatic HEYA8 (HEYA8 NM) cells (Fig. 5A). These findings suggest an inverse correlation between miR-199a-3p expression and chemoresistance. Next, we evaluated if miR-199a-3p can alter chemosensitivity of ovarian cancer cells. Ectopic expression of miR-199a-3p could sensitize A2780/DDP to cisplatin and SKOV-3 cells to cisplatin and paclitaxel (Fig. 5B, C). To further connect our findings to response to chemotherapy, a clinical cohort of high-grade serous ovarian carcinoma patients’ samples was assessed. In accordance with our earlier findings, platinum-resistant patient tumors also had a lower expression of miR-199a-3p as compared to platinum-sensitive ones (Fig. 5D). Moreover, low miR-199a-3p expression was able to independently predict progression-free survival (Fig. 5E). These results indicate that miR-199a-3p expression is inversely related to chemoresistance of ovarian cancer and a poor clinical outcome.

### miR-199a-3p overexpression reduces metastasis and sensitizes them to chemotherapeutics in an in vivo xenograft model of ovarian cancer

**Fig. 6:**
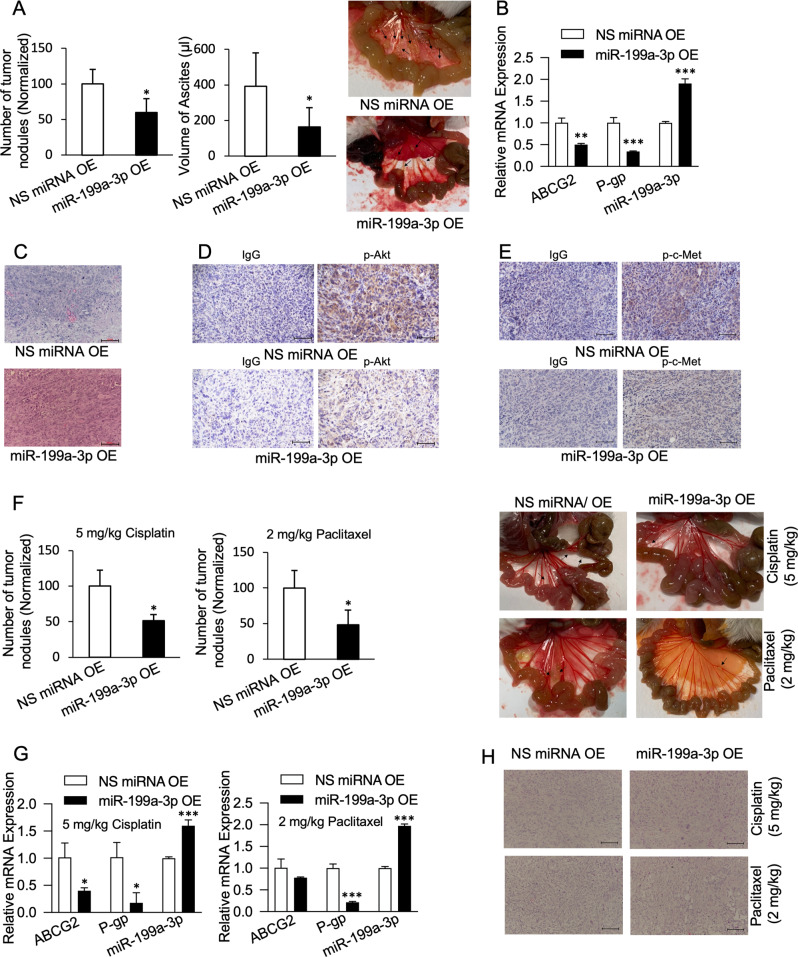
miR-199a-3p reduces peritoneal dissemination of SKOV-3 cells and sensitizes them to cisplatin and paclitaxel. **A** Number of tumor nodules, volume of ascites, and representative image of tumor nodules. **B** Expression of ABCG2, P-gp, and miR-199a-3p from tumor tissue samples. **C** H&E, **D** p-Akt, and **E** p-c-Met staining of tumor tissue samples. **F** Number of tumor nodules and representative images for cisplatin and paclitaxel treated group. **G** ABCG2, P-gp, and miR-199a-3p expression, and **H** H&E staining after cisplatin/paclitaxel treatment. Scale bar, 100 μm. Bar graph values are mean ± SD, *n* = 3. *T*-test. *P < 0.05, **P < 0.005, ***P < 0.0005.

To assess the potential therapeutic role of miR-199a-3p in ovarian cancer, we established SKOV-3 cells stably overexpressing miR-199a-3p using a lentiviral vector for miR-199a-1. The expression of miR-199a-1 and miR-199a-3p in these cells was verified by qPCR (Supplementary Fig. 2). Upon injection in NOD/SCID mice, mice with miR-199a-3p-overexpressing cells exhibited lower volume of ascites and number of metastatic nodules in the peritoneal cavity than non-specific miRNA control (Fig. 6A). These results demonstrated that miR-199a-3p reduced the peritoneal dissemination of tumor cells. Moreover, tumors harvested from mice had significantly lower mRNA expression of chemoresistance markers ABCG2 and P-gp for the miR-199a-3p-overexpressing group (Fig. 6B). Similarly, immunohistochemical staining showed lower levels of p-c-Met and p-Akt in tumors overexpressing miR-199a-3p (Fig. 6C-E).

Next, we evaluated the role of miR-199a-3p in response to cisplatin and paclitaxel treatment. miR-199a-3p-overexpressing tumors were significantly more susceptible to both cisplatin and paclitaxel, and they harbored lower expression of chemoresistance markers ABCG2 and P-gp (Fig. 6F–H). We also performed these experiments by stably expressing miR-199a-3p in A2780/DDP cells and observed similar results (Supplementary Fig. 3). Together, these data suggest that miR-199a-3p suppresses peritoneal dissemination of ovarian cancer cells and could effectively sensitize them to the mainstay chemotherapeutics cisplatin and paclitaxel.

## Discussion

Ovarian cancer has an extremely poor prognosis and there is a lack of drug targets. This paper presents two novel and important findings. First, we show that biophysical and biochemical cues in the ascitic tumor microenvironment can concertedly regulate miR-199a-3p. This effect is mediated via a previously unknown transcriptional regulation of miR-199a-3p through a c-Met/PI3K/Akt-signaling pathway in a positive feedback loop to enhance tumor aggressiveness. Second, we demonstrate the potential importance of miR-199a-3p as a novel prognostic marker for ovarian cancer. Thus, the combination of miR-199a-3p and chemotherapeutic drugs could be of great therapeutic value.

HGF and its receptor c-Met are involved in malignant transformation [30], and c-Met overexpression in ovarian cancer is a prognostic factor [31]. Although ovarian cancer cells exhibit high expression of c-Met, they are reliant on the ascitic microenvironment for HGF [32, 33]. Intriguingly, here we found that shear stress stimulation in the absence of additional HGF could also activate c-Met, demonstrating that c-Met is a shear stress-responsive tyrosine kinase receptor. Although other receptor tyrosine kinases such as caveolin-1 and Tie2 are previously known to be activated by shear stress [34, 35], these studies have been carried out in devices modeling shear stress in the blood vessel [6]. It is noteworthy that fluid flow in the peritoneum is significantly different from that in blood vessels, and tumor cells experience several folds lower magnitude of shear stress in the peritoneal cavity. Since the magnitude of shear stress can determine the biological outcome, the finding that c-Met is a low shear stress-activated protein is of particular relevance to peritoneal dissemination of tumor cells. The ascitic microenvironment contains high levels of HGF, which promotes metastasis and invasiveness of ovarian cancer through activation of c-Met. The HGF-independent activation of c-Met by shear stress in the peritoneal cavity may explain why HGF-neutralizing antibodies alone are only partially effective in inhibiting metastasis [36, 37]. On the other hand, the concerted regulation of miR-199a-3p by shear stress and HGF may explain the emergence of chemoresistant tumors in the late stages of ovarian cancer.

After their discovery in the 1990s, miRNAs were quickly recognized as critical modulators of gene expression and essential regulators of signaling in carcinogenesis. Studies on the upstream biogenesis of miRNAs showed that this largely involves post-transcriptional processing mediated by Dicer, Drosha and Exportin-5. Although there are many studies have looked into downstream targets of miR-199a-3p, its regulation is not well understood [38]. In this study, we found that shear stress did not affect the processors of miRNA biogenesis. Instead, we have identified a previously unknown transcriptional regulation of miR-199a-3p. These observations are in line with our previous study which suggested that shear stress does not induce global but rather change in a specific miRNA expression [23]. Transcriptional dysregulation is a common feature of cancers, and alteration of cell signaling through it determines the phenotypic outcome [39]. Transcriptional changes tend to induce more long-lasting effect than post transcriptional and other cellular changes, and thus represent a more actionable mechanism. Previous studies in endothelial cells have revealed shear stress regulation of miRNAs but they mainly involve post-transcriptional mechanisms [40, 41]. This could possibly be due to the difference in the shear stress magnitudes between the peritoneal cavity (at least 10-fold lower than that of vascular shear stress) and blood vessels. Delineating context-specific regulation of miRNA is critical for identifying specific targets. Since shear stress specifically alters the transcription of miR-199a-3p without affecting the processors of miRNA biogenesis, targeting miR-199a-3p is likely to result in abrogation of the biological consequence of shear stress without affecting other members of the miRNA network.

Shear stress-mediated activation of c-Met led to signaling through PI3K/Akt, a well-known pathway involved in chemoresistance in the clinic. Activation of PI3K is found in around 70% of ovarian cancers [8], and our findings here suggest that ascitic shear stress is a causative factor. There are several inhibitors targeting PI3K/Akt signaling in ovarian cancer, but it remains to be explored how PI3K/Akt induces chemoresistance. Our findings here provide a novel mechanistic insight into the involvement of this pathway in acquiring resistance through downregulation of miR-199a-3p, which may be beneficial in drug designing and other clinical applications in the future.

The vast majority of ovarian cancer cases are detected after the tumor has metastasized, making cisplatin and paclitaxel the first-line treatment modality for patients. However, as tumors become more resistant, the increase in chemotherapy dosage leads to complications and discontinuation of treatment [42]. Here we show that low expression on miR-199a-3p is involved in driving the stem-like properties in ovarian cancer cells. It is also hypothesized that stemness in cancer cells is associated with chemoresistant properties [43]. Intriguingly, overexpression of miR-199a-3p could diminish tumor initiation capabilities of ovarian cancer cells and sensitize them to clinically relevant doses of cisplatin and paclitaxel. Although there are no currently approved miRNA-based therapeutics, initial trials are showing promising results. In this study we have demonstrated the utility of miR-199a-3p as an indicator of disease prognosis and could thus serve as a biomarker for ovarian cancer. In the clinic, miR-199a-3p mimics could therefore sensitize tumors to chemotherapy, allowing continuation of treatment and improved quality of life for ovarian cancer patients.

Taken together, our findings provide evidence for the first time that c-Met is a shear stress-responsive receptor tyrosine kinase and provides mechanistic insight into its downstream regulation of miR-199a-3p through a positive feedback loop between c-Met/PI3K/Akt/miR-199a-3p. To the best of our knowledge, this demonstrates a novel cooperation between biophysical and biochemical cues in the ascitic microenvironment. Moreover, we provide a direct causal and potentially druggable link between this that also explains stemness and chemoresistance in ovarian cancer. Notably, the delineated pathway specifically regulates the functionally relevant component, miR-199a-3p, which represents a viable target for efficacious therapeutic development.

## Materials and methods

### Cells and cell culture

Human ovarian cancer cell lines SKOV-3 and A2780 were kind gifts from Dr. N. Auersperg (University of British Columbia, Canada). HEY A8 cells were a gift from Dr. J. Liu (MD Anderson Cancer Center, USA). SKOV-3 cells were maintained in Medium 199: MCDB 105 in 1:1 ratio supplemented with 5% fetal bovine serum (Hyclone). A2780 and HEYA8 cells were maintained in in Roswell Park Memorial Institute (RPMI) medium supplemented with 10% and 5% FBS, respectively. All cells were maintained in 1% penicillin and streptomycin (100 units/ml) at 37 °C under 5% CO_2_. The cell lines were tested for mycoplasma contamination by PCR as described previously [44], and cell line identities were verified by Pangenia Inc (Hong Kong) by short tandem repeat (STR) analysis. For microfluidic chip experiments, SKOV-3 cells were cultured as described previously [23]. Briefly, 96-well plates coated with 0.5% agarose were seeded at a concentration of 1000 cells/well. After 48 h, spheroids were collected and washed thrice with serum-free media and loaded into the microfluidic chip with a pipette tip (approximately 60 spheroids per channel).

### Patient tumor samples

Patient tumor samples were obtained from ovarian cancer patients (Stages I–IV) undergoing surgery at Shuang Ho Hospital (Taipei, Taiwan) with informed consent. The specimens were snap-frozen immediately and stored at −80 °C. The presence of tumor cells was verified by histological examination by pathologists. The age of patients ranged from 26 to 83, with a mean age of 58.5. Further details of patient stratification are shown in Fig. 5D. The usage of samples was approved by the Taipei Medical University Institutional Review Board.

### Microfluidic chip experiments

Chips containing three channels each of 4 mm width, 25 mm length, and 250 μm height were manufactured as described previously [23]. Spheroids were stimulated at shear stress of 0.02 dyne/cm^2^ by introducing fluid flow at 301.2 μl/h via a multi-syringe pump (LongerPump) connected to the channel with a gas-permeable silicone tube (Scientific Commodities Inc.). To prevent spheroid attachment, the channels were coated with 12 mg/ml poly 2-hydroxyethylmethacrylate (Sigma-Aldrich). Wall shear stress was calculated using the following equation:

$$\tau = \frac{{6Q{\upmu}}}{{h^2w}}$$

where *τ* is the wall shear stress; *Q* is the flow rate; *μ* is the viscosity (0.01 g cm s^−1^); *h* is the height of channel; and *w* is the width of channel.

### RNA extraction, reverse transcription, and quantitative real-time PCR (qPCR)

Cells were lysed with TRIzol (Invitrogen Life Technologies, Inc.) and total RNA was extracted following manufacturer’s protocol. Reverse transcription of mature miRNA was carried out with TaqMan MicroRNA Reverse Transcription Kit (ThermoScientific) using the following miRNA-specific stem-loop primers at a final concentration of 50 nM: U6, 5′-GTCGTATCCAGTGCAGGGTCCGAGGTATTCGCACTGGATACGACAAAAAATAT-3′; miR-199a-3p, 5′-CTCAACTGGTGTCGTGGAGTCGGCAATTCAGTTGAGTAACCAAT-3′. For cDNA synthesis from mRNA and primary miRNA, 1 μg total RNA was reverse transcribed with oligo dT primers and gene-specific primers respectively using MMLV-RT (Invitrogen). For primary miRNAs, cDNA was synthesized using a cocktail of the following gene specific primers at 20 nM each: U1, 5′-AGGGGAAAGCGCGAACG-3′, miR-199a-1, 5′-GGAAAATGACACTCACC-3′; miR-199a-2, 5′-CCCTAGTGTGCAAAACC-3′. Negative RT controls were used to exclude possible amplification from genomic DNA. Quantitative real-time PCR analysis was performed in ABI StepOnePlusTM Real-Time PCR System (Applied Biosystems) using Aceq qPCR SYBR Green Master Mix (Vazyme Biotech) with gene-specific primers at a final concentration of 0.2 μM. Quantification of gene expression was performed using the delta-delta Ct method (2^–∆∆Ct^). The following are the primer sequences: miR-199a-3p, 5′-ACACTCCAGCTGGGACAGTAGTCTGCACAT-3′ (forward) and 5′-GTGGAGTCGGCAATTC-3′ (reverse); DICER1, 5′-GTGCTGCAGTAAGCTGTGCTA-3′ (forward) and 5′-TGCTGAAGTCTCCCCTGATCT-3′ (reverse); DROSHA, 5′-GATCTGGAAGTCGCTCCCCAAG-3′ (forward) and 5′-ATGGTCTCCTCGGGCTCTTT-3′ (reverse); XPO5, 5′-TGGCCACAGAGGTCACCCCC-3′ (forward) and 5′-GGGGCGCAGTGCCTCGTAT-3′ (reverse); miR-199a-1, 5′-CGACGAGCCGCTCACCCAAG-3′ (forward) and 5′-GTGGTGGTTTCCTTGGCT-3′ (reverse); miR-199a-2, 5′-GGAGGCTTTTCCTGAGGAC-3′ (forward) and 5′-CCCTAGTGTGCAAAACCTGT-3′ (reverse); MET, 5′ATACGGTCCTATGGCTGGTG-3′ (forward) and 5′-TTGAGAGGTTCTTTCCACCAAGT-3′ (reverse); CD44, 5′-CCTCACATCCAACACCTCCC-3′ (forward) and 5′-TGGTTGCTGTCTCAGTTGCT-3′ (reverse); ALDH3A1, 5′-GCAGCAGTTCGGCTTAGGA-3′ (forward) and 5′-CTAGGACGTACACCACCTCC-3′ (reverse); ABCG2, 5′-CTGAGATCCTGAGCCTTTGG-3′ (forward) and 5′-TGCCCATCACAACATCATCT-3′ (reverse); ABCB1 (P-gp), 5′-GAGCCTACTTGGTGGCACAT-3′ (forward) and 5′-TCCTTCCAATGTGTTCGGCA-3′ (reverse); GAPDH, 5′-GGAGCGAGATCCCTCCAAAAT-3′ (forward) and 5′-GGCTGTTGTCATACTTCTCATGG-3′ (reverse). Internal controls for mature miRNA, mRNA, and primary miRNA were U6, GAPDH, and U1, respectively.

### Western blot

Cells were lysed in sodium dodecyl sulfate lysis buffer and denatured by boiling in a final concentration of 0.1 M dithiothreitol. The samples were subjected to sodium dodecyl sulfate–polyacrylamide gel electrophoresis (SDS-PAGE) and transferred to nitrocellulose membrane (Bio-Rad). Membranes were blocked in 5% non-fat milk in phosphate buffered saline (PBS) containing 0.05% Tween-20 (PBST) and incubated with anti-p-Akt (Cell Signaling Technology, Catalog No. 4060), anti-Total Akt (Cell Signaling Technology, Catalog No. 4691), anti-p-c-Met (Cell Signaling Technology, Catalog No. 3077), anti-Total c-Met (Cell Signaling Technology, Catalog No. 3127), or anti-β-actin (Sigma-Aldrich, Catalog No. A5316) primary antibody overnight at 4 °C. Appropriate horseradish peroxidase-conjugated secondary antibodies were used for detection of bands.

### siRNA and miRNA transfection

Cells were seeded in six-well plates 24 h before transfection. 20 nM Dicer siRNA (Dharmacon, Catalog No. D-003483), Drosha siRNA (Dharmacon, Catalog No. D-016996), Exportin-5 siRNA (Dharmacon, Catalog No. D-014000), Akt siRNA (Dharmacon, Catalog No. D-003000), or c-Met siRNA (Dharmacon, Catalog No. L-003156) was transfected with 1.5 μl siLectFect (Bio-Rad) for 24 h. Similar protocol was used for transfecting miR-199a-3p mimic (Ambion, Catalog No. 4464066) at 10 nM concentration. Cells were at 80% confluence at the time of transfection. Respective non-specific controls were incorporated in each experimental setup.

### Stable overexpression of miRNA

Lentivirus for miR-199a-1 was purchased from (BioSettia, Catalog No. mir-LV156) at a titer of 10^7^ IU/ml. SKOV-3 cells stable overexpressing Luciferase were transduced with 20 μl lentivirus. Cells successfully transduced were selected with 1 μg/ml puromycin (Calbiochem) over 7 days.

### Spheroid formation assay

For spheroid formation assay, cells were seeded at a concentration of 5000 cells/ml in a low adhesion dish in serum free media. The number of spheroids formed after 5 days was counted using a bright field microscope (Nikon, Tokyo, Japan).

### Chemosensitivity assay

Chemosensitivity was assayed using 3-(4,5-dimethylthiazol-2-yl)-2,5-diphenyl tetrazolium bromide (MTT). After treatment with chemotherapeutic reagent, cells/spheroids were incubated in 20 μl 5 mg/ml MTT solution (in PBS) for 2–4 h at 37 °C. The MTT solution was aspirated, and the resulting formazan salt was resuspended in DMSO. Readings were taken at 570 nm wavelength using a spectrophotometer (Bio-Rad Microplate reader Model 550, Hercules, CA). For experiments involving cisplatin, relative cell viability was calculated as percentage over control after subtracting blank. For paclitaxel experiments, cell viability was calculated relative to maximum response.

### RNA sequencing and in silico analysis

Total RNA extracted from shear stress-treated cells were sent to Beijing Genomics Institute for RNA sequencing. Differential expression between genes were determined by log_2_-fold difference in fragments per kilobase million (FPKM) values of samples. For overrepresentation analysis, list of genes with >1.5-fold expression in shear stress compared to stationary culture was analyzed using EnrichR.

### In vivo xenograft model

4 × 10^6^ SKOV-3 cells stably overexpressing miR-199a-3p or non-specific control were intraperitoneally injected in female NOD/SCID mice of 4–6 weeks. After allowing 4 weeks for tumor growth, cisplatin and paclitaxel was injected intraperitoneally twice a week at 5 mg/kg for 1 week and 2 mg/kg for 3 weeks, respectively. Animals were randomly allocated into groups of 3. Extent of tumor metastasis was evaluated by counting number of tumor nodules formed in the peritoneal cavity and the volume of ascites formed at the time of sacrifice. Tumor tissues were harvested and fixed in for further downstream analysis. All animal experiments were carried out according to institution-approved protocols in compliance with the Committee for the Use of Laboratory Animals at the University of Hong Kong.

### Statistical analysis

Statistical analysis was performed using GraphPad Prism (GraphPad Prism, San Diego, CA, USA). Data were presented as mean ± standard deviation (SD). Student’s one-tailed *t* test was used to determine statistical significance between test groups for qPCR relative quantification. For multiple groups, multiples *t*-tests were performed against control. Significance of platinum sensitivity of patient tumors was determined by Fisher’s Exact Test. *P < 0.05, **P < 0.005, ***P < 0.0005 was considered statistically significant.

## Supplementary information

Supplemental Figures

Original Data File

Checklist

## Data Availability

All relevant data are included in the article and Supplementary materials. Additional data are available from the corresponding author upon reasonable request.
