# Supplementary material for: Ascitic fluid shear stress in concert with hepatocyte growth factor drive stemness and chemoresistance of ovarian cancer cells via the c-Met-PI3K/Akt-miR-199a-3p signaling pathway
Source: Cell Death Dis. 2022 Jun 8;13(6):537. doi: 10.1038/s41419-022-04976-6 (PMC9177676; doi:10.1038/s41419-022-04976-6)
Supplement: Supplementary file 1 — Supplemental Figures [file 41419_2022_4976_MOESM1_ESM.pptx]

## Slide 1
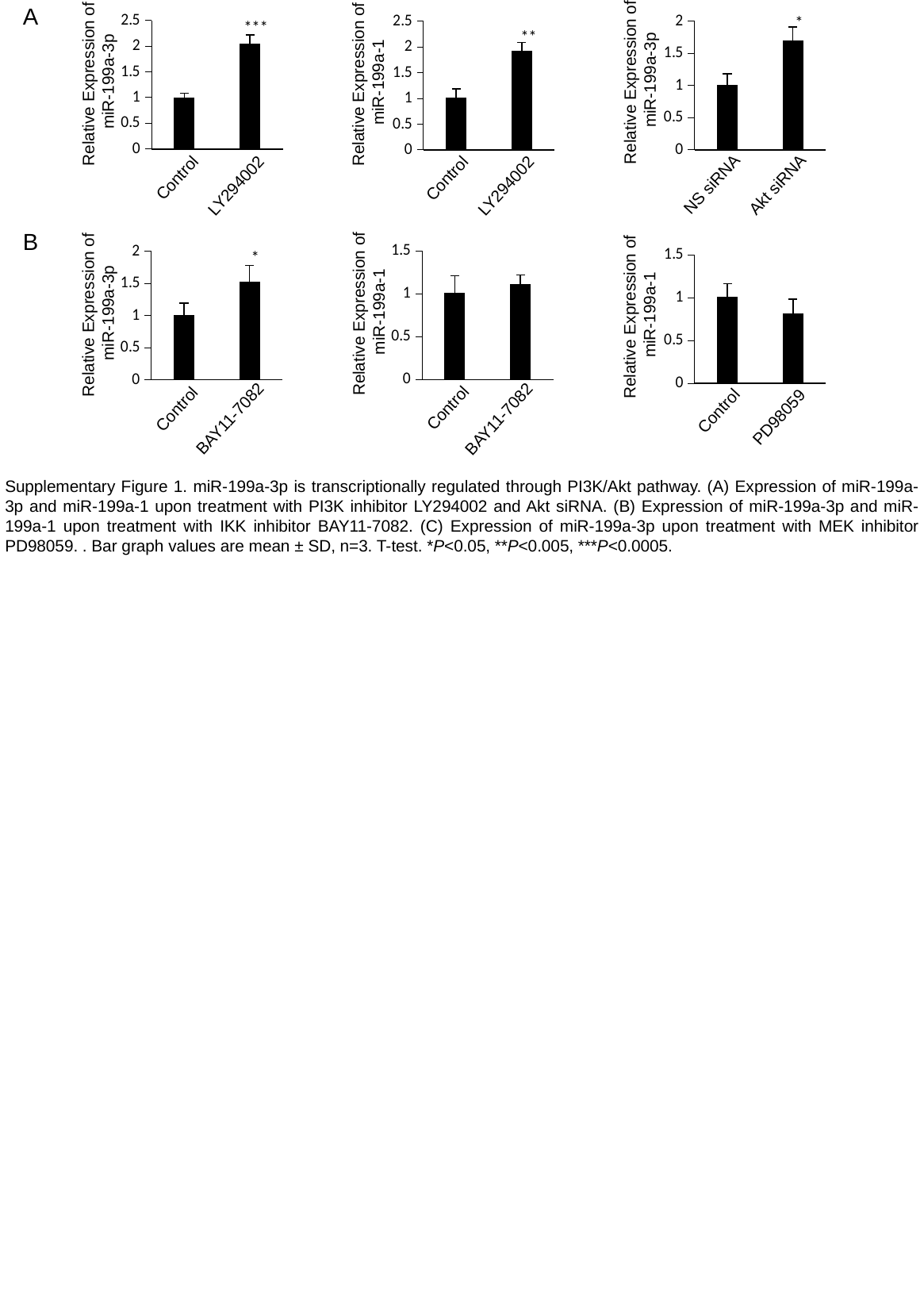

### Chart
| Category | |
|---|---|Relative Expression of
miR-199a-3p
Control
LY294002
***
### Chart
| Category | |
|---|---|**
Relative Expression of
miR-199a-1
Control
LY294002
### Chart
| Category | |
|---|---|*
Relative Expression of
miR-199a-3p
NS siRNA
Akt siRNA
A
### Chart
| Category | |
|---|---|Relative Expression of
miR-199a-1
Control
BAY11-7082
### Chart
| Category | |
|---|---|*
Relative Expression of
miR-199a-3p
Control
BAY11-7082
### Chart
| Category | |
|---|---|Relative Expression of
miR-199a-1
Control
PD98059
B
Supplementary Figure 1. miR-199a-3p is transcriptionally regulated through PI3K/Akt pathway. (A) Expression of miR-199a-3p and miR-199a-1 upon treatment with PI3K inhibitor LY294002 and Akt siRNA. (B) Expression of miR-199a-3p and miR-199a-1 upon treatment with IKK inhibitor BAY11-7082. (C) Expression of miR-199a-3p upon treatment with MEK inhibitor PD98059. . Bar graph values are mean ± SD, n=3. T-test. *P<0.05, **P<0.005, ***P<0.0005.

## Slide 2
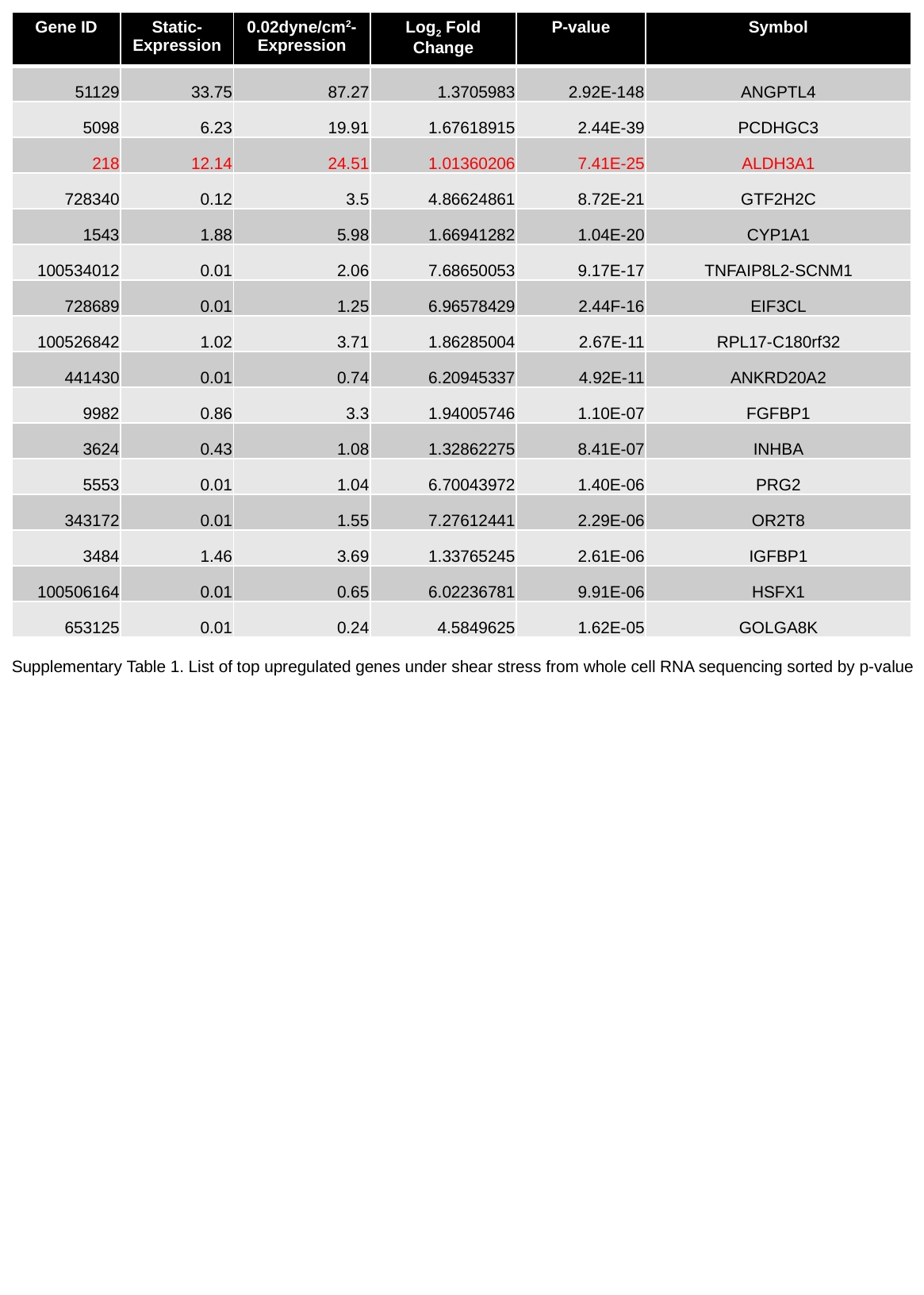

| Gene ID | Static-Expression | 0.02dyne/cm2-Expression | Log2 Fold Change | P-value | Symbol |
| --- | --- | --- | --- | --- | --- |
| 51129 | 33.75 | 87.27 | 1.3705983 | 2.92E-148 | ANGPTL4 |
| 5098 | 6.23 | 19.91 | 1.67618915 | 2.44E-39 | PCDHGC3 |
| 218 | 12.14 | 24.51 | 1.01360206 | 7.41E-25 | ALDH3A1 |
| 728340 | 0.12 | 3.5 | 4.86624861 | 8.72E-21 | GTF2H2C |
| 1543 | 1.88 | 5.98 | 1.66941282 | 1.04E-20 | CYP1A1 |
| 100534012 | 0.01 | 2.06 | 7.68650053 | 9.17E-17 | TNFAIP8L2-SCNM1 |
| 728689 | 0.01 | 1.25 | 6.96578429 | 2.44F-16 | EIF3CL |
| 100526842 | 1.02 | 3.71 | 1.86285004 | 2.67E-11 | RPL17-C180rf32 |
| 441430 | 0.01 | 0.74 | 6.20945337 | 4.92E-11 | ANKRD20A2 |
| 9982 | 0.86 | 3.3 | 1.94005746 | 1.10E-07 | FGFBP1 |
| 3624 | 0.43 | 1.08 | 1.32862275 | 8.41E-07 | INHBA |
| 5553 | 0.01 | 1.04 | 6.70043972 | 1.40E-06 | PRG2 |
| 343172 | 0.01 | 1.55 | 7.27612441 | 2.29E-06 | OR2T8 |
| 3484 | 1.46 | 3.69 | 1.33765245 | 2.61E-06 | IGFBP1 |
| 100506164 | 0.01 | 0.65 | 6.02236781 | 9.91E-06 | HSFX1 |
| 653125 | 0.01 | 0.24 | 4.5849625 | 1.62E-05 | GOLGA8K |
Supplementary Table 1. List of top upregulated genes under shear stress from whole cell RNA sequencing sorted by p-value

## Slide 3
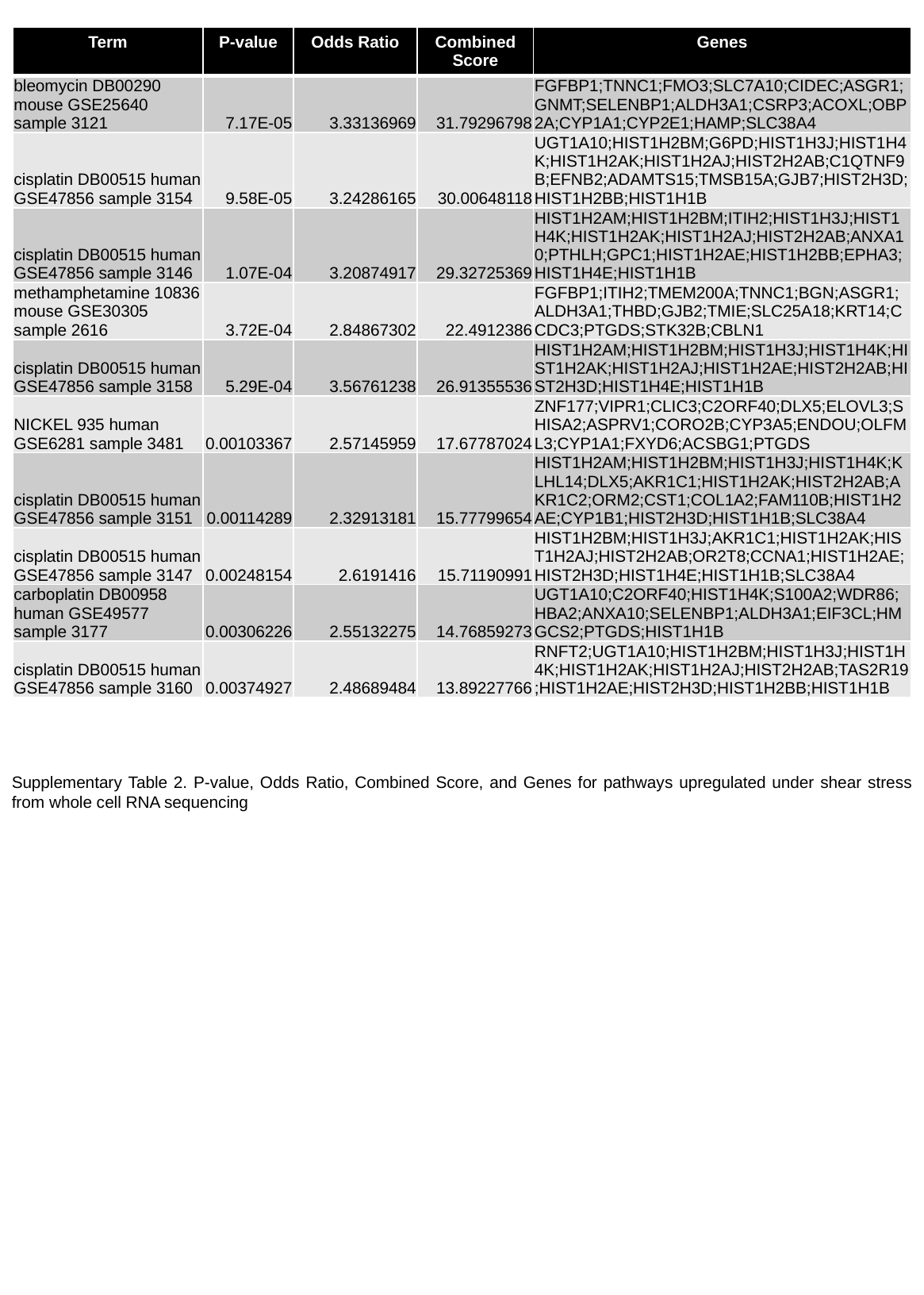

| Term | P-value | Odds Ratio | Combined Score | Genes |
| --- | --- | --- | --- | --- |
| bleomycin DB00290 mouse GSE25640 sample 3121 | 7.17E-05 | 3.33136969 | 31.79296798 | FGFBP1;TNNC1;FMO3;SLC7A10;CIDEC;ASGR1;GNMT;SELENBP1;ALDH3A1;CSRP3;ACOXL;OBP2A;CYP1A1;CYP2E1;HAMP;SLC38A4 |
| cisplatin DB00515 human GSE47856 sample 3154 | 9.58E-05 | 3.24286165 | 30.00648118 | UGT1A10;HIST1H2BM;G6PD;HIST1H3J;HIST1H4K;HIST1H2AK;HIST1H2AJ;HIST2H2AB;C1QTNF9B;EFNB2;ADAMTS15;TMSB15A;GJB7;HIST2H3D;HIST1H2BB;HIST1H1B |
| cisplatin DB00515 human GSE47856 sample 3146 | 1.07E-04 | 3.20874917 | 29.32725369 | HIST1H2AM;HIST1H2BM;ITIH2;HIST1H3J;HIST1H4K;HIST1H2AK;HIST1H2AJ;HIST2H2AB;ANXA10;PTHLH;GPC1;HIST1H2AE;HIST1H2BB;EPHA3;HIST1H4E;HIST1H1B |
| methamphetamine 10836 mouse GSE30305 sample 2616 | 3.72E-04 | 2.84867302 | 22.4912386 | FGFBP1;ITIH2;TMEM200A;TNNC1;BGN;ASGR1;ALDH3A1;THBD;GJB2;TMIE;SLC25A18;KRT14;CCDC3;PTGDS;STK32B;CBLN1 |
| cisplatin DB00515 human GSE47856 sample 3158 | 5.29E-04 | 3.56761238 | 26.91355536 | HIST1H2AM;HIST1H2BM;HIST1H3J;HIST1H4K;HIST1H2AK;HIST1H2AJ;HIST1H2AE;HIST2H2AB;HIST2H3D;HIST1H4E;HIST1H1B |
| NICKEL 935 human GSE6281 sample 3481 | 0.00103367 | 2.57145959 | 17.67787024 | ZNF177;VIPR1;CLIC3;C2ORF40;DLX5;ELOVL3;SHISA2;ASPRV1;CORO2B;CYP3A5;ENDOU;OLFML3;CYP1A1;FXYD6;ACSBG1;PTGDS |
| cisplatin DB00515 human GSE47856 sample 3151 | 0.00114289 | 2.32913181 | 15.77799654 | HIST1H2AM;HIST1H2BM;HIST1H3J;HIST1H4K;KLHL14;DLX5;AKR1C1;HIST1H2AK;HIST2H2AB;AKR1C2;ORM2;CST1;COL1A2;FAM110B;HIST1H2AE;CYP1B1;HIST2H3D;HIST1H1B;SLC38A4 |
| cisplatin DB00515 human GSE47856 sample 3147 | 0.00248154 | 2.6191416 | 15.71190991 | HIST1H2BM;HIST1H3J;AKR1C1;HIST1H2AK;HIST1H2AJ;HIST2H2AB;OR2T8;CCNA1;HIST1H2AE;HIST2H3D;HIST1H4E;HIST1H1B;SLC38A4 |
| carboplatin DB00958 human GSE49577 sample 3177 | 0.00306226 | 2.55132275 | 14.76859273 | UGT1A10;C2ORF40;HIST1H4K;S100A2;WDR86;HBA2;ANXA10;SELENBP1;ALDH3A1;EIF3CL;HMGCS2;PTGDS;HIST1H1B |
| cisplatin DB00515 human GSE47856 sample 3160 | 0.00374927 | 2.48689484 | 13.89227766 | RNFT2;UGT1A10;HIST1H2BM;HIST1H3J;HIST1H4K;HIST1H2AK;HIST1H2AJ;HIST2H2AB;TAS2R19;HIST1H2AE;HIST2H3D;HIST1H2BB;HIST1H1B |
Supplementary Table 2. P-value, Odds Ratio, Combined Score, and Genes for pathways upregulated under shear stress from whole cell RNA sequencing

## Slide 4
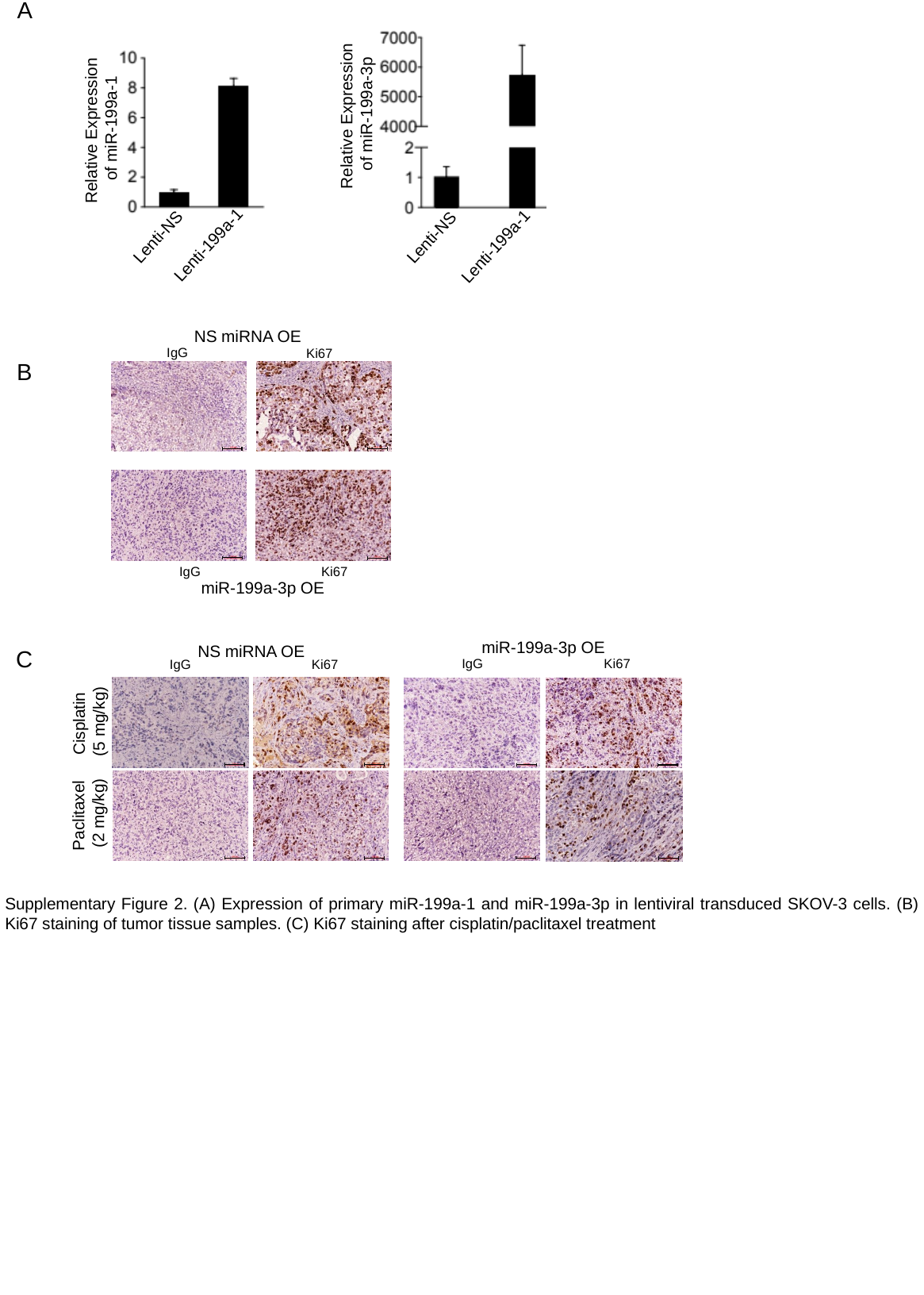

A
Relative Expression
of miR-199a-3p
Lenti-NS
Lenti-199a-1
Relative Expression
of miR-199a-1
Lenti-NS
Lenti-199a-1
NS miRNA OE
IgG
Ki67
IgG
Ki67
miR-199a-3p OE
B
miR-199a-3p OE
NS miRNA OE
IgG
Ki67
IgG
Ki67
Cisplatin
(5 mg/kg)
Paclitaxel
(2 mg/kg)
C
Supplementary Figure 2. (A) Expression of primary miR-199a-1 and miR-199a-3p in lentiviral transduced SKOV-3 cells. (B) Ki67 staining of tumor tissue samples. (C) Ki67 staining after cisplatin/paclitaxel treatment

## Slide 5
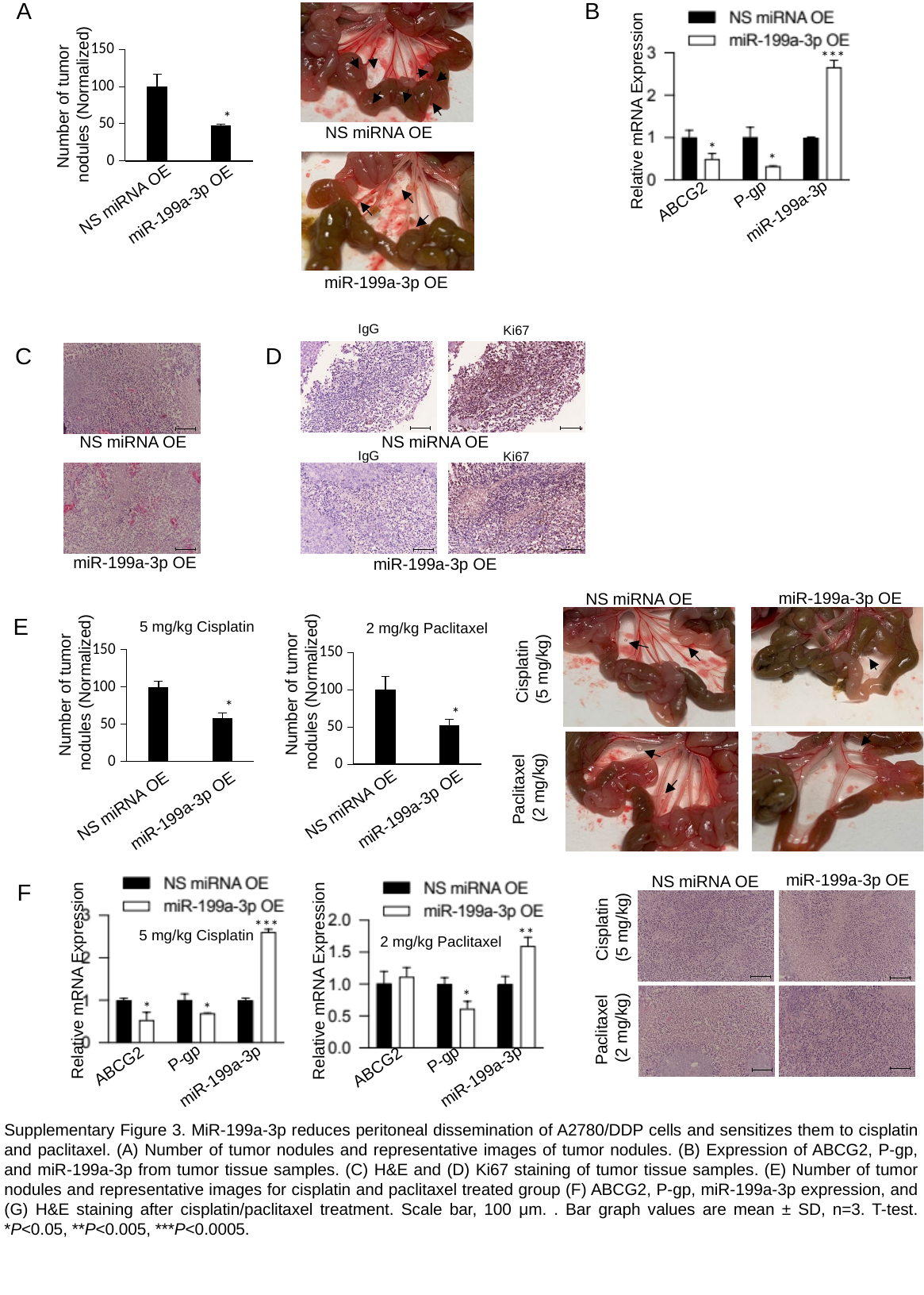

A
B
Relative mRNA Expression
miR-199a-3p
P-gp
ABCG2
***
*
*
NS miRNA OE
miR-199a-3p OE
### Chart
| Category | |
|---|---|Number of tumor
nodules (Normalized)
NS miRNA OE
miR-199a-3p OE
*
IgG
Ki67
C
D
NS miRNA OE
miR-199a-3p OE
NS miRNA OE
IgG
Ki67
miR-199a-3p OE
miR-199a-3p OE
NS miRNA OE
Cisplatin
(5 mg/kg)
Paclitaxel
(2 mg/kg)
2 mg/kg Paclitaxel
Number of tumor
nodules (Normalized)
NS miRNA OE
miR-199a-3p OE
### Chart
| Category | |
|---|---|*
5 mg/kg Cisplatin
### Chart
| Category | |
|---|---|Number of tumor
nodules (Normalized)
NS miRNA OE
miR-199a-3p OE
E
*
miR-199a-3p OE
NS miRNA OE
Cisplatin
(5 mg/kg)
Paclitaxel
(2 mg/kg)
***
5 mg/kg Cisplatin
Relative mRNA Expression
*
*
P-gp
miR-199a-3p
ABCG2
2 mg/kg Paclitaxel
Relative mRNA Expression
*
miR-199a-3p
P-gp
ABCG2
F
**
Supplementary Figure 3. MiR-199a-3p reduces peritoneal dissemination of A2780/DDP cells and sensitizes them to cisplatin and paclitaxel. (A) Number of tumor nodules and representative images of tumor nodules. (B) Expression of ABCG2, P-gp, and miR-199a-3p from tumor tissue samples. (C) H&E and (D) Ki67 staining of tumor tissue samples. (E) Number of tumor nodules and representative images for cisplatin and paclitaxel treated group (F) ABCG2, P-gp, miR-199a-3p expression, and (G) H&E staining after cisplatin/paclitaxel treatment. Scale bar, 100 μm. . Bar graph values are mean ± SD, n=3. T-test. *P<0.05, **P<0.005, ***P<0.0005.
