# Supplementary material for: Ascitic fluid shear stress in concert with hepatocyte growth factor drive stemness and chemoresistance of ovarian cancer cells via the c-Met-PI3K/Akt-miR-199a-3p signaling pathway
Source: Cell Death Dis. 2022 Jun 8;13(6):537. doi: 10.1038/s41419-022-04976-6 (PMC9177676; doi:10.1038/s41419-022-04976-6)

This file contains original data of Western Blot in the manuscript. The images were obtained as .tif files using Bio-Rad ChemiDoc Imaging System

Figure 3b

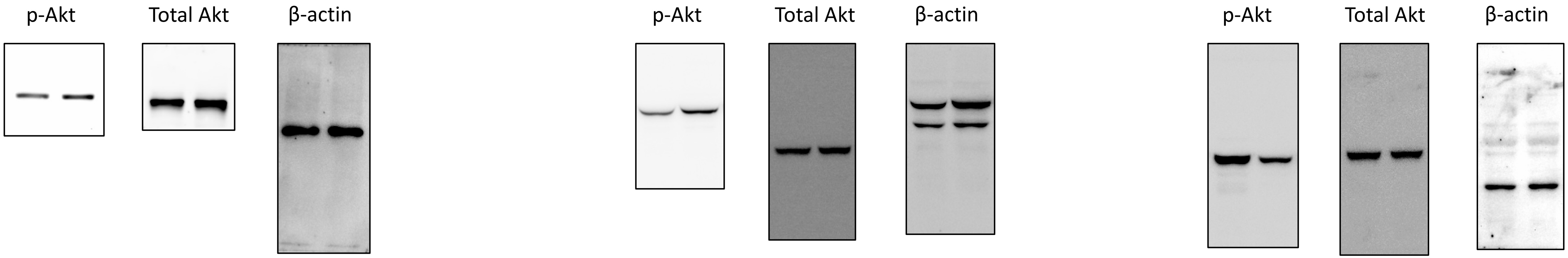
p-Akt
Total Akt
β-actin
p-Akt
Total Akt
β-actin
p-Akt
Total Akt
β-actin

Figure 3d

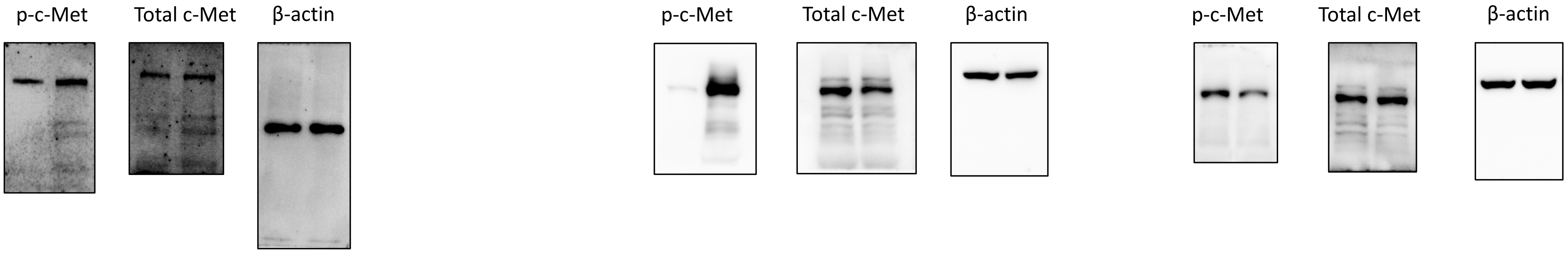
p-c-Met
Total c-Met
β-actin
p-c-Met
Total c-Met
β-actin
p-c-Met
Total c-Met
β-actin

Figure 3f

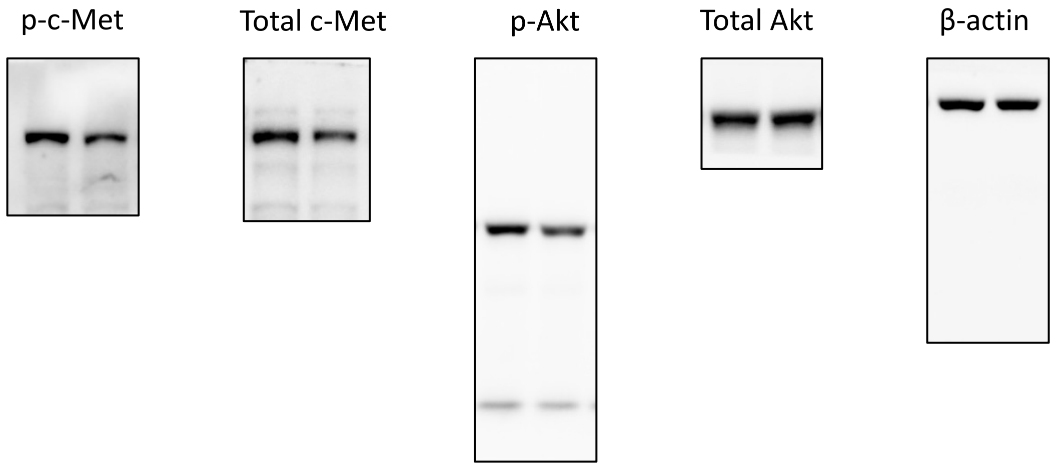

Supplement: Supplementary file 2 — Original Data File [file 41419_2022_4976_MOESM2_ESM.pdf]
